# First Manic Episode Following SARS-CoV-2 Infection

**DOI:** 10.7759/cureus.33986

**Published:** 2023-01-20

**Authors:** Bedir Alihsan, Simon Kashfi, Dennis T Roarke

**Affiliations:** 1 Internal Medicine, Zucker School of Medicine at Hofstra/Northwell Health, Manhasset, USA

**Keywords:** manic episodes, mental health and covid-19, covid and mental health, covid-19 mental health, coronavirus, first manic episode, neuropsychiatric, sars-cov-2, covid 19, mania

## Abstract

Since the beginning of the COVID-19 pandemic, there have been reports of neuropsychiatric symptoms following infection with the severe acute respiratory syndrome coronavirus 2 (SARS-CoV-2), most notably mania and psychosis. However, despite the widely reported incidence of psychosis and mania following infection with SARS-CoV-2, a causal link between the virus and these neuropsychiatric symptoms has not been established. A myriad of confounding factors such as underlying psychiatric disorders, personal and family psychiatric histories, substance use, and treatment with steroids all have the ability to obscure a correlation between SARS-CoV-2 and subsequent psychiatric symptoms. Here we present a case of a manic episode in a 40-year-old male following a COVID-19 infection. He had no past psychiatric history, no family psychiatric history, and no history of substance use. This case is unique in that the patient lacks all these typical confounding variables. It should serve as an example of a first-time manic episode following a recent infection with SARS-CoV-2. It may contribute data to future investigations seeking to better elucidate the correlation between SARS-CoV-2 and neuropsychiatric symptoms such as mania.

## Introduction

Since its discovery in the autumn of 2019, the severe acute respiratory syndrome coronavirus 2 (SARS-CoV-2) and its associated disease, coronavirus disease 2019 (COVID-19), have caused a myriad of associated symptoms. The majority of these take the form of respiratory symptoms; however, neuropsychiatric symptoms have also been widely reported [1]. The most common of these is anosmia; however, other neuropsychiatric manifestations of COVID-19 have also been reported, notably psychosis and mania. It has been known since early in the pandemic that the SARS-CoV-2 virus is able to enter the central nervous system, as it has been found in the cerebral spinal fluid (CSF) of patients with active COVID-19 infections [2]. However, the consequences or exact mechanisms by which the virus accomplishes this have yet to be established. Early in the course of the pandemic, case reports of COVID-19 patients developing manic symptoms emerged [3]. Of course, viral infection, severe illness, and subsequent hospitalization are all psychologically stressful events that can exacerbate underlying psychiatric disorders like bipolar disorder. However, there have been multiple reports of first-time manic episodes occurring in the setting of a SARS-CoV-2 infection and in the absence of any previous psychiatric history [4]. This has brought into question whether these manic symptoms are in fact a neuropsychiatric manifestation of COVID-19 itself. We present a 40-year-old patient with no past psychiatric history or family psychiatric history who presented with a manic episode shortly after a SARS-CoV-2 infection.

## Case presentation

A 40-year-old African American male with no personal psychiatric history and no psychiatric history among any of his immediate family members was admitted to our hospital in the northeastern United States. Collateral history also did not report any mental illness or previous diagnosis. The patient was a highly successful individual with multiple academic accomplishments and a prestigious job in education. He had no history of a substance use disorder, reporting only a distant history of cannabis use from years prior. His only known past medical history is minor mitral regurgitation, diagnosed when he was a teenager. He had taken no medications and reported no allergies. Three weeks prior, the patient contracted COVID-19 from his daughter and was diagnosed by a polymerase chain reaction (PCR) test at a local urgent care center. He had received three vaccines, though it is unknown which specific brand he received. He had minimal symptoms, including fever, cough, sore throat, rhinorrhea, and fatigue. He took no medication to treat his symptoms during this time and chose to isolate himself in his home. His initial symptoms began to resolve after five days of infection; however, the patient’s wife reported that around this time the patient had a decreased need for sleep and became more talkative.

Over the course of the next two weeks, the patient became increasingly outgoing, loquacious, and motivated, especially regarding his work. Initially, his colleagues welcomed his heightened enthusiasm; however, in the days leading up to the patient’s admission, both his family and friends became concerned with his behavior. At a staff meeting, the patient spoke verbosely, recounting his life story, making odd gestures, and removing several articles of clothing. He displayed increasingly goal-directed behavior, proposing projects for his workplace that became excessively ambitious and complex. This behavior prompted his wife to take him to his primary care physician, who in turn recommended that the patient go to the emergency department out of concern for his mental status. At the time of admission, twenty days after being diagnosed with COVID-19, the patient had not slept in ten days. He had a normal sleeping pattern prior to contracting the infection.

On examination, the patient displayed signs of grandiosity, claiming that he knew more about medicine than any physician in the hospital. When asked how he was feeling, he attempted to enter a discussion with his examiner about drainage basins, river deltas, and game theory and then proceeded to remove his clothes and perform headstands in the hallway, refusing to cooperate with hospital staff until he was given a private room with the temperature set to exactly 65 degrees. He reported no auditory or visual hallucinations and denied suicidal or homicidal ideation. He was not responding to internal stimuli. The patient was oriented to time, place, person, and situation. He had poor judgment and insight into his condition. The patient was admitted to a general medical floor for the management of a manic episode, with the possibility of involuntary inpatient psychiatric hospitalization if he did not improve.

During his stay, the patient underwent a thorough medical and neurological examination to rule out any underlying causes of his mania. The workup involved general labs such as a complete blood count, comprehensive metabolic panel, iron studies, venous blood gas, urine toxicology screen, urinalysis, thyroid function tests, erythrocyte sedimentation rate, and C-reactive protein. He also received an extensive infectious workup, including blood cultures, urine cultures, a tick-borne illness panel, a viral hepatitis panel, HIV testing, and a respiratory viral panel. Other studies included heavy metal levels, including mercury, arsenic, cadmium, and lead; vitamin levels; an ammonia level; a blood alcohol level; an alpha-beta T-cell receptor; and a double-negative T-cell count and percentage. A chest X-ray, non-contrast computed tomography brain scan, and brain magnetic resonance image with and without contrast were also performed. All studies were grossly normal and unable to identify an organic cause of the patient’s manic symptoms.

The patient was started on valproate and olanzapine, which were titrated up to 1250 mg/day and 10 mg/day, respectively. The patient displayed manic symptoms for the first four days of his hospital stay, after which he was finally able to sleep for seven hours overnight. On his fifth day of admission, the patient displayed improved symptoms. His grandiosity and irritability had resolved. He remained distractable with pressured speech but was easily redirectable by the physicians managing him. By the sixth day of admission, the patient's only remaining manic symptom was minor pressured speech, which resolved the next day. He was discharged after seven days of inpatient treatment on valproic acid and olanzapine. His valproic acid level the day before discharge was 68 ug/mL (normal range: 50-100 ug/mL). Upon discharge, the patient was back to his baseline. At a follow-up appointment one week later, he felt normal and asked his provider to wean him off his psychiatric medications.

## Discussion

Neuropsychiatric symptoms are not uncommon manifestations of viral infections. Cognitive and motor impairment are well documented in cases of human immunodeficiency virus, and hepatitis C is known to cause depression, anxiety, and "brain fog" [5,6]. Mania, in particular, is a complex psychiatric syndrome that is most commonly associated with bipolar disorder; however, a variety of other factors have been associated with mania, such as substance use, genetics, treatment with corticosteroids, and viral infection [7]. Manic symptoms presenting during an active SARS-CoV-2 infection have been reported since early in the pandemic [3]. There is growing evidence that COVID-19 and psychiatric diagnoses have a complex bidirectional relationship, each acting as an independent risk factor for the other [8]. A recent review, however, discovered evidence of multiple cases in which a patient with no known past psychiatric history was diagnosed with a first manic episode shortly after the SARS-CoV-2 infection [9].

Mania in the context of COVID-19 is a complex topic with many confounding factors obscuring its study. The illness itself has enormous potential to serve as a trigger for an underlying bipolar disorder that is yet to be diagnosed. In the above-mentioned systematic review, almost every patient included had a history of psychiatric illness in a first-degree relative [9]. Although much is still unknown about bipolar disorder and mania, a strong genetic component has been identified [10]. A first-degree relative with a history of bipolar disorder remains one of the strongest risk factors for developing a diagnosis of bipolar disorder. Some studies have shown patients are 7.9 times more likely to be diagnosed with bipolar disorder if they have a first-degree relative with a bipolar diagnosis [11]. Additionally, the most current treatment of COVID-19 involves some use of corticosteroids, most commonly dexamethasone, a well-established trigger of mania, which further obfuscates the etiology of the manic episode [12].

This case serves as a unique example in which all of these potential confounding factors were absent. The patient was at no increased risk for the development of mania: he had no family history of psychiatric disorders, he had not been treated with corticosteroids for his recent SARS-CoV-2 infection, and furthermore, he was at an unusual age for the development of bipolar disorder. The average age of bipolar diagnosis, usually via a first manic episode, is 20 years old [13]. Based on the Diagnostic and Statistical Manual of Mental Disorders (DSM)-V criteria, his symptoms were consistent with all criteria for a manic episode [14]. He had an extensive medical workup that failed to identify another cause of his manic symptoms. This case is unique in that the only abnormality in the patient’s psychiatric or medical history is a recent infection with SARS-CoV-2.

Although mania has sometimes been reported in the context of COVID-19 infection, the pathophysiology is far from elucidated. Hypotheses are being developed that take into account factors such as inflammation, changes in genetics and epigenetics, metabolism, hormones, neurotransmission, immunology, and oxidative stress [15]. Even though the virus has been shown to invade the central nervous system, a clear clinical correlation between positive CSF PCR testing and neuropsychiatric symptoms has not been identified [16]. As previously mentioned, a host of confounding factors impede the study of mania in COVID patients. Another recent systematic review identified 21 additional cases of a first manic episode without a diagnosis of bipolar disorder following the SARS-CoV-2 infection [4]. The study adds further evidence to the correlation between manic episodes and COVID-19; however, the sample size was low. Although manic and psychotic symptoms have been reported following SARS-CoV-2 infections, investigators lack adequate data to sufficiently study the issue. This case should stand as an example of possible SARS-CoV-2-induced mania, further adding to the growing evidence that there is a correlation between COVID-19 and manic symptoms.

## Conclusions

Neuropsychiatric symptoms, specifically mania and psychosis, following infection with SARS-CoV-2 have been reported since the beginning of the pandemic. Although SARS-CoV-2 has been demonstrated to enter the CSF, the clinical significance of this has not been established. Manic symptoms occurring in the context of COVID-19 are difficult to study given a variety of compounding factors, including past psychiatric history, psychological stressors of COVID-19, treatment with steroids, and substance use. This case demonstrates a patient with no predisposing risk factors for bipolar disorder, no psychiatric family history, and no substance use who presented with a first-time manic episode shortly after an otherwise uncomplicated infection with SARS-CoV-2. He was treated with olanzapine and valproate and recovered after a seven-day hospital stay that did not require inpatient psychiatric admission. Future investigations should attempt to include more cases without confounding variables to better elucidate the correlation and potential mechanisms between SARS-CoV-2 infection and neuropsychiatric symptoms such as mania.
